# The Need for Device-Specific Training Before Handheld Ultrasound Use: Agreement and Repeatability of Simulated Aortic Aneurysm Diameter Measurements by Senior Sonography Students on a Familiar Conventional and an Unfamiliar Handheld System

**DOI:** 10.3390/diagnostics16213524

**Published:** 2026-10-27

**Authors:** Mahasin G. Hassan, Dema Alenazi, Salsabil Aldosari, Alanoud Alotaibi, Rema Alrajhi, Lamis Al-Sultan, Mohammed Alsaadi

**Affiliations:** 1 Department of Radiological Sciences, College of Health and Rehabilitation Sciences, Princess Nourah bint Abdulrahman University, P.O. Box 84428, Riyadh 11671, Saudi Arabia; 444000527@pnu.edu.sa (D.A.); 444000811@pnu.edu.sa (S.A.); 444000868@pnu.edu.sa (A.A.); 444001022@pnu.edu.sa (R.A.); 443000210@pnu.edu.sa (L.A.-S.); 2 Radiology and Medical Imaging Department, College of Applied Medical Sciences in Al-Kharj, Prince Sattam Bin Abdulaziz University, Al-Kharj 11942, Saudi Arabia; m.alsaadi@psau.edu.sa

**Keywords:** abdominal aortic aneurysm, ultrasound, handheld ultrasound, measurement agreement, Bland–Altman, intraclass correlation coefficient, phantom, sonography education, device-specific training, operator experience

## Abstract

**Background:** Ultrasound measurement of abdominal aortic aneurysm (AAA) diameter guides screening and surveillance, but whether students’ measurement competence on a conventional system transfers to an unfamiliar handheld device has rarely been assessed. **Methods:** Twenty-seven senior sonography students made three anteroposterior outer-to-outer measurements of two water-filled balloons embedded in gelatin (reference diameters 3.53 and 5.88 cm, calculated from the circumference before embedding), first on the familiar cart-based conventional system and then, in a fixed, non-randomised order, on a handheld system with which they had no prior hands-on experience. The primary outcomes were bias against the calculated reference, with equivalence testing (±5%), and the paired between-system difference, with Bland–Altman analysis; repeatability was assessed with repeatability coefficients. **Results:** Conventional measurements were equivalent to the calculated reference within ±5% (bias −0.09 cm, 95% CI −0.16 to −0.02; +0.07 cm, 95% CI −0.05 to +0.19). Handheld measurements were higher (+0.17 and +0.33 cm; both *p* < 0.001) and failed equivalence; the mean between-system difference was +0.26 cm (95% CI 0.18–0.34), without proportional bias. Single-reading repeatability coefficients were 0.31–0.67 cm. **Conclusions:** Students’ conventional-system measurements agreed closely with the calculated reference, although this reference was not verified after embedding. Because device type was confounded with operator inexperience and with a fixed testing order, which introduces an unquantifiable learning or fatigue effect, the 0.26 cm value is an observed between-system difference under the study conditions and cannot be attributed to the handheld device itself. Measurement competence did not transfer automatically to a new device, underlining the critical need for device-specific training before handheld systems are used for aortic measurement. These implications are educational and technical; phantom findings do not support clinical surveillance recommendations without confirmation in patients.

## 1. Introduction

An abdominal aortic aneurysm (AAA) is conventionally defined as a maximum infrarenal aortic diameter of 3.0 cm or more, or a focal dilatation exceeding 1.5 times the diameter of the adjacent normal segment [1]. The Global Burden of Disease analysis attributed approximately 170,000 deaths per year to AAA and estimated a global prevalence of 0.92% among adults aged 30–79 years, rising steeply after 60 years of age and higher in men than in women [2]. Because more than 80% of aneurysms are asymptomatic until rupture, ultrasound screening of at-risk men has been shown to reduce aneurysm-related mortality, and one-time ultrasound screening is recommended by the U.S. Preventive Services Task Force and by European vascular societies for men aged 65 years and older, smokers and individuals with a positive family history [3,4].

Once an aneurysm is detected, maximum aortic diameter, together with its rate of growth, is the principal variable that determines management [5,6,7]. Current guidelines recommend surveillance intervals of approximately three years for aneurysms of 3.0–3.9 cm, one year for 4.0–4.9 cm and three to six months for 5.0–5.4 cm, with consideration of elective repair at 5.5 cm in men and 5.0 cm in women, or earlier if the aneurysm grows rapidly or becomes symptomatic [5,6]. These thresholds are separated by only a few millimetres, so a measurement error of 3–5 mm can change a surveillance interval or the timing of referral for further assessment. In current practice, ultrasound is the principal modality for screening and serial surveillance, whereas computed tomography angiography (CTA), which is less operator-dependent, provides three-dimensional anatomical detail and avoids the calliper-placement ambiguities of ultrasound, is the accepted standard for pre-operative planning and for the definitive determination of eligibility for repair [5,6,8]. The practical consequence of an ultrasound measurement error is thus not a direct change in the operative decision but an inappropriate surveillance interval or a premature or delayed referral for CTA. Measurement accuracy and reproducibility are therefore central concerns in ultrasound practice and training [9].

Several studies have shown that novices can acquire aortic scanning competence after short, structured training. Nguyen et al. found that non-physician trainees could be taught to screen for AAA with high agreement against expert readers [10], Bonnafy et al. reported that novice operators using a pocket-sized system achieved acceptable reliability for aortic diameter [11], and medical students have been shown to detect AAA with point-of-care ultrasound as well as, or better than, physical examination by vascular surgeons [12] and to measure aortic dimensions accurately with handheld devices in primary care [13].

Nevertheless, ultrasound measurement of the aorta is subject to multiple sources of variability, including operator training and experience, the ultrasound system and transducer frequency, body habitus, calcification and mural thrombus, aortic tortuosity, image plane, measurement axis, calliper placement convention, the level at which the diameter is taken and the phase of the cardiac cycle [14,15]. Calliper placement alone has a substantial effect: outer-to-outer (OTO), inner-to-inner (ITI) and leading-edge-to-leading-edge (LELE) conventions differ systematically, with mean differences of approximately 4–5 mm between OTO and ITI readings, and the ITI and LELE methods have shown better inter-observer reproducibility than OTO in multicentre studies [16,17,18]. In the UK national screening programme, inter-observer reproducibility coefficients of the anteroposterior diameter have been reported in the range of 4–6 mm, and the cardiac cycle contributes a further 1–2 mm of variation [19,20].

Handheld ultrasound devices add a further layer of variability. A systematic review of handheld versus high-end systems concluded that agreement is generally good for abdominal applications, including AAA detection, but that image quality and measurement precision remain lower on handheld devices [21]. Technical assessments have confirmed that the spatial resolution of handheld probes is inferior to cart-based systems, with potential implications for calliper placement on vessel walls [22], and medical students have reported reduced image quality on handheld compared with standard machines [23]. These differences reflect, at least in part, hardware design: cart-based consoles generally have more processing channels and dedicated hardware beamforming, which provide higher spatial resolution and signal-to-noise ratio [21,22]. At the same time, performance on any device depends on the operator’s familiarity with its image optimisation and calliper interface, so device-related and operator-related sources of error are difficult to separate when operators are more experienced with one system than with another.

Sonography students in the study institution complete dedicated courses in ultrasound physics, abdominal ultrasound, vascular ultrasound and quality control before their final year, but the extent to which this training translates into aortic measurements that agree closely with a reference value and are repeatable, particularly on handheld devices that are not routinely used in their clinical placements, has not been evaluated. Phantom studies allow this question to be addressed against a reference diameter determined independently of ultrasound rather than one inferred from another imaging modality, under controlled conditions that remove patient-related variability. By the same token, phantoms do not reproduce the acoustic challenges of clinical scanning, such as body habitus, bowel gas and mural thrombus, so their results describe baseline operator and equipment performance rather than clinical accuracy [24].

The aims of the present study were therefore (i) to quantify the bias and limits of agreement of AAA diameter measurements made by senior sonography students, relative to the pre-embedding calculated diameter of two balloon-in-gelatin phantoms, on a conventional and a handheld ultrasound system; (ii) to quantify the intra-observer repeatability of these measurements on each system; and (iii) to determine whether the two systems differ systematically from each other and whether error depends on aneurysm size. Because the students had used only the conventional system during their training, the study was designed to examine whether measurement competence transfers to an unfamiliar handheld device, not to establish the intrinsic accuracy of either device. We hypothesised that measurements would be closer to the reference on the familiar conventional system and that any between-system difference would be systematic rather than random.

## 2. Materials and Methods

### 2.1. Study Design, Setting and Participants

This was a quantitative, cross-sectional method-comparison study in which each participant measured two phantoms with two ultrasound systems in a single session. The study was conducted between January and May 2026 in the ultrasound laboratory of the Department of Radiological Sciences at the study institution in Riyadh, Saudi Arabia.

The target population was the entire cohort of senior (Level 7 and Level 8) undergraduate students enrolled in the Radiological Sciences programme during the 2025–2026 academic year who had completed all core ultrasound courses (Ultrasound Physics, Abdominal Ultrasound, Vascular Ultrasound and Quality Control), including their theoretical, laboratory and clinical components. This cohort was chosen because it provides a homogeneous training background at the transition to clinical practice. Internship students were excluded because of heterogeneity in clinical exposure and supervision; students from other institutions, students who had not completed all core ultrasound courses, students who were unavailable or unwilling to participate, and members of the research team were also excluded. Total population sampling of the eligible cohort yielded 27 participants, all of whom completed the full protocol with no missing data.

### 2.2. Phantoms and Pre-Embedding Calculated Reference Diameter

Two tissue-mimicking phantoms were constructed from bovine gelatin with psyllium (Isabgol) husk powder as a scattering agent, following the general approach of gelatin-based training phantoms [24,25] and the gelatin–psyllium formulation whose acoustic properties and shelf life have been characterised previously [26,27]. Twelve 10 g sachets of commercial bovine gelatin were used for each phantom. For the first layer, five sachets were dissolved in 430 mL of boiling water with one tablespoon of psyllium husk, strained to remove lumps and poured into a rectangular plastic mould. When this layer had partially set, a water-filled latex balloon simulating the aneurysm was positioned on its surface. The second layer (seven sachets in 590 mL of water with 1.5 tablespoons of psyllium husk) was strained and poured over the balloon to cover it completely, and the phantom was left to solidify. Phantoms were kept in closed containers at room temperature (22–25 °C) between sessions. The phantoms were designed to provide a reference diameter determined independently of ultrasound under controlled conditions. Each is a simplified model, a water-filled balloon embedded in gelatin, and does not reproduce the wall structure, tortuosity or irregular morphology of an aneurysmal aorta; the term “phantom” in this manuscript refers to this simplified model. The phantoms have an approximately uniform speed of sound and lack subcutaneous fat, bowel gas, phase aberration, acoustic clutter, pulsatility, calcification and mural thrombus, and therefore do not replicate clinical scanning conditions (Section 4.7).

**Figure 1:**
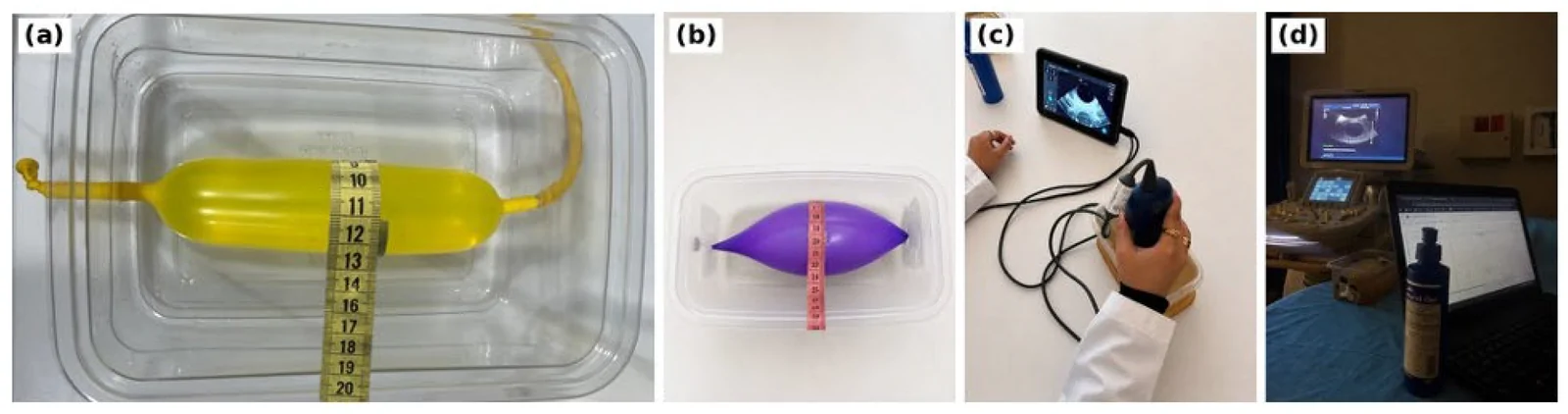
Phantoms and experimental setup. (**a**) Phantom A (reference diameter 3.53 cm) and (**b**) Phantom B (reference diameter 5.88 cm) before embedding, showing circumference measurement of the water-filled balloon; (**c**) a participant measuring a phantom with the handheld Kosmos device; (**d**) the conventional Philips iU22 workstation used for the same measurement.

The reference diameter of each balloon was calculated before embedding by measuring its circumference at the widest point with a flexible tape (resolution 0.1 cm) and applying D = C/π. A 0.1 cm error in circumference therefore corresponds to a 0.03 cm error in the reference diameter. This calculation assumes a circular cross-section and that the balloon geometry did not change after embedding, whereas the participants measured the anteroposterior diameter of the embedded balloon; the two quantities are therefore not necessarily identical. The reference is accordingly referred to as the pre-embedding calculated reference diameter, and the closeness of measurements to it is described as “agreement” rather than “accuracy”, because it is not a directly verified true diameter. The two phantoms were deliberately constructed to represent the two clinically distinct size categories (Figure 1a,b):

- Phantom A (reference diameter 3.53 cm): An aneurysm within the surveillance range (3.0–5.4 cm) that does not require intervention but is clinically significant;
- Phantom B (reference diameter 5.88 cm): An aneurysm above the diameter thresholds at which elective repair is considered in both men (5.5 cm) and women (5.0 cm according to the ESVS 2024 guideline and 5.0–5.4 cm according to the SVS 2018 guideline) [5,6].

The reference diameters were not disclosed to the participants. Because the reference value was derived from the balloon before embedding and the phantoms were used over a five-month data-collection period, phantom stability was examined post hoc by testing for a temporal trend in measured diameter across the sequence of participant sessions (Section 2.5). This analysis can detect a change in the phantoms over time but cannot validate the absolute value of the reference.

### 2.3. Ultrasound Systems

The conventional system was a Philips iU22 cart-based diagnostic ultrasound system (Philips Healthcare, Bothell, WA, USA) with a C5-1 curvilinear transducer (1–5 MHz) operated with the abdominal preset (Figure 1d). The handheld system was the Kosmos point-of-care ultrasound platform (EchoNous, Redmond, WA, USA) with the Kosmos Torso probe (1.5–4.5 MHz) operated with the abdominal preset on its tablet display (Figure 1c) [28]. Both systems provide electronic callipers for linear distance measurement. Participants had used the conventional system routinely during their clinical placements but had no prior hands-on experience with the handheld device beyond a brief orientation to its controls immediately before the session. Device type was therefore confounded with operator familiarity: the study compares measurements made on a familiar system with those made on an unfamiliar one and was not designed to establish the intrinsic measurement accuracy of either device.

### 2.4. Measurement Protocol

All measurements were obtained in the transverse plane at the widest point of the simulated aneurysm, along the anteroposterior (AP) dimension, using the outer-to-outer (OTO) calliper convention, which is the convention taught in the programme’s vascular ultrasound course. Each participant obtained three independent measurements of each phantom with each system, re-placing the transducer and re-acquiring the image before each measurement, giving 12 measurements per participant (2 phantoms × 2 systems × 3 repetitions) and 324 measurements in total. The order of phantoms and systems was the same for all participants (conventional system first, then handheld; Phantom A then Phantom B). This order was not randomised or counterbalanced, so device type was also confounded with measurement order, and any learning or fatigue effect accumulating during the session could not be quantified or separated from the between-system difference (Section 4.7). Measurements were read from the on-screen callipers to two decimal places (0.01 cm) and recorded in a spreadsheet by a member of the research team; participants were not shown their previous values or those of other participants. The mean of the three readings was the unit of analysis for agreement and between-system comparisons, while the three individual readings were used for the repeatability analysis.

### 2.5. Statistical Analysis

Statistical analyses were performed using IBM SPSS Statistics version 32.0 (IBM Corp., Armonk, NY, USA). Two-sided *p* < 0.05 was considered statistically significant. The primary outcomes were (i) the bias of each system relative to the calculated reference, with its 95% CI and the equivalence test, and (ii) the paired between-system difference, with its 95% CI and Bland–Altman limits of agreement. The within-participant SD and the repeatability coefficient were the main secondary outcomes. All other analyses (non-parametric tests; MAD, MAPE and RMSE; error categories and McNemar tests; repeated-measures ANOVA and Friedman tests; ICCs; threshold-crossing counts; and phantom-stability regressions) were exploratory or supportive, were not adjusted for multiplicity and are interpreted descriptively. Throughout, “agreement” refers to the closeness of measurements to the calculated reference or between the two systems (bias and limits of agreement), “repeatability” to the consistency of repeated readings by the same participant on the same system, and “reproducibility” to the variation between participants measuring the same phantom under identical conditions.

*Descriptive statistics and normality.* Per-participant means are summarised as mean ± standard deviation (SD) with 95% confidence intervals (CIs), median with interquartile range (IQR) and range. Normality of the per-participant means and of the paired differences was assessed with the Shapiro–Wilk test and inspection of histograms and Q–Q plots. When normality was rejected, we reported a non-parametric test (Wilcoxon signed-rank test) alongside the parametric test; conclusions did not differ between them.

*Agreement with the calculated reference.* For each phantom–system condition, bias was defined as the mean of (measured−reference) with its 95% CI and tested with a one-sample *t*-test (and Wilcoxon signed-rank test). Because a non-significant difference from the reference cannot by itself be interpreted as agreement, equivalence was additionally tested with the two one-sided tests (TOST) procedure [29], computed from the one-sample *t*-test output, using a pre-specified margin of ±5% of the reference diameter (±0.18 cm for Phantom A; ±0.29 cm for Phantom B), which corresponds to the ≤5% band labelled “accurate” in vascular imaging [30]. That source uses the 5% band to classify diameter agreement and does not validate it as an equivalence margin for phantom measurements. The margin was chosen because, in absolute terms (0.18 and 0.29 cm), it is smaller than the inter-observer variability of approximately 0.4–0.6 cm reported for clinical aortic ultrasound [9,15,19] and smaller than the 3–5 mm measurement error that can change a surveillance interval (Section 1), so that a bias within this margin would be small relative to the measurement error tolerated in practice. Because any margin involves judgement, the 90% CI of each bias, which shows the narrowest margin at which equivalence would be established, is also reported. Agreement with the reference was described by Bland–Altman 95% limits of agreement (LoA; bias ± 1.96 SD of the differences) [31,32]. Because the reference is a fixed value rather than a second method subject to random measurement error, differences were plotted against the reference value rather than against the mean of the two methods, as recommended by Krouwer [33]. Mean absolute deviation (MAD), mean absolute percentage error (MAPE) and root-mean-square error (RMSE) were also computed. Each participant’s mean was classified by its absolute percentage difference from the calculated reference as ≤5%, >5% to ≤10% or >10% [30], and the proportion of participants in each category was compared between systems with an exact McNemar test. The number of participant means that crossed a guideline surveillance or intervention threshold (4.0 cm for Phantom A; 5.5 cm for Phantom B) was also counted as an illustrative indicator; because the phantoms do not reproduce clinical scanning conditions, this count is not intended as an estimate of clinical misclassification.

*Comparison between systems.* The paired difference (handheld−conventional) was analysed with a paired *t*-test (and Wilcoxon signed-rank test), the standardised effect size dz, and Bland–Altman analysis with 95% CIs for the bias and for the LoA [32]; proportional bias was tested by regressing the differences on the mean of the two systems. A two-way repeated-measures ANOVA with system (conventional, handheld) and phantom (A, B) as within-subject factors was fitted to the signed percentage error, to test whether the between-system difference depended on aneurysm size, and to the absolute percentage error, to test whether the magnitude of the relative error depended on aneurysm size; generalised eta-squared (ηG2) is reported as the effect size.

*Repeatability (intra-observer reliability)*. Reliability of the three repeated readings was quantified with the intraclass correlation coefficient computed from a two-way random-effects, absolute-agreement model, reported for a single reading, ICC(2,1), and for the mean of three readings, ICC(2,3), with 95% CIs, following the Shrout–Fleiss/McGraw–Wong formulation and the reporting guidance of Koo and Li [34]. Conventional ICC reliability categories (poor to excellent) were not applied, because in this design the between-participant variance does not reflect true variation in aneurysm size (see below); the absolute indices were treated as the primary measures of repeatability. It should be noted that in this design every participant measured the same object, so the between-participant variance that enters the ICC reflects participant-specific offsets rather than true between-subject variation in aortic size; the ICC therefore quantifies how consistently each participant reproduces his or her own reading relative to the spread among participants and should be read alongside the absolute indices. These were the within-subject SD (SDw, from one-way ANOVA), the within-subject coefficient of variation, and the repeatability coefficient (1.96 × √2 × SDw), which is the expected upper limit of the absolute difference between two readings by the same operator [31,32]. Between-participant variability under identical conditions was summarised by the SD of the participant means and the corresponding reproducibility coefficient (1.96 × √2 × SDbetween) [9]; a conventional inter-observer ICC could not be estimated because only two targets (phantoms) were measured. Cronbach’s α was not used as a reliability index because it measures the internal consistency of multi-item scales, is insensitive to systematic differences between readings, and is not an appropriate measure of agreement for repeated physical measurements [35].

*Phantom stability.* As a check on the temporal stability of the phantoms over the data-collection period, the per-participant mean diameter was regressed on the participant sequence number (participants were enrolled and identifiers assigned in chronological order of the measurement session) for each phantom–system condition, and the first nine and last nine participants were compared with an independent-samples *t*-test. A gelatin phantom that dehydrated or a balloon that lost water would be expected to produce a negative trend.

*Power.* No formal a priori sample-size calculation was possible because the eligible cohort was fixed and enrolled in full. A sensitivity power analysis was therefore performed: with n = 27 paired observations, α = 0.05 (two-sided) and 80% power, the minimum detectable standardised paired effect is dz = 0.56 (0.65 for 90% power) [36]. Using the observed SDs, this corresponds to a minimum detectable bias of approximately 0.10 cm (Phantom A) and 0.17–0.18 cm (Phantom B) against the calculated reference and to a minimum detectable between-system difference of 0.11 cm (Phantom A) and 0.21 cm (Phantom B). Achieved (post hoc) power for the observed effects is not reported, because it adds no information beyond the effect estimates and their 95% CIs, which are used to judge precision.

## 3. Results

### 3.1. Participants and Data

All 27 eligible senior students participated and completed the protocol, yielding 324 individual readings and 108 per-participant means (27 participants × 2 phantoms × 2 systems). No readings were missing or excluded. The Shapiro–Wilk test did not reject normality of the participant means for Phantom A with the conventional system (W = 0.979, *p* = 0.84) or Phantom B with the handheld device (W = 0.942, *p* = 0.13), but did for Phantom A with the handheld device (W = 0.884, *p* = 0.006; positively skewed by three participants with means > 3.9 cm) and Phantom B with the conventional system (W = 0.901, *p* = 0.014; one participant with a mean of 6.98 cm). Paired between-system differences were normally distributed for Phantom A (W = 0.987, *p* = 0.98) but not for Phantom B (W = 0.918, *p* = 0.035). Non-parametric tests were therefore performed alongside parametric tests; in every case they led to the same conclusion, and their *p*-values are given in the text or table footnotes.

### 3.2. Descriptive Statistics

**Figure 2:**
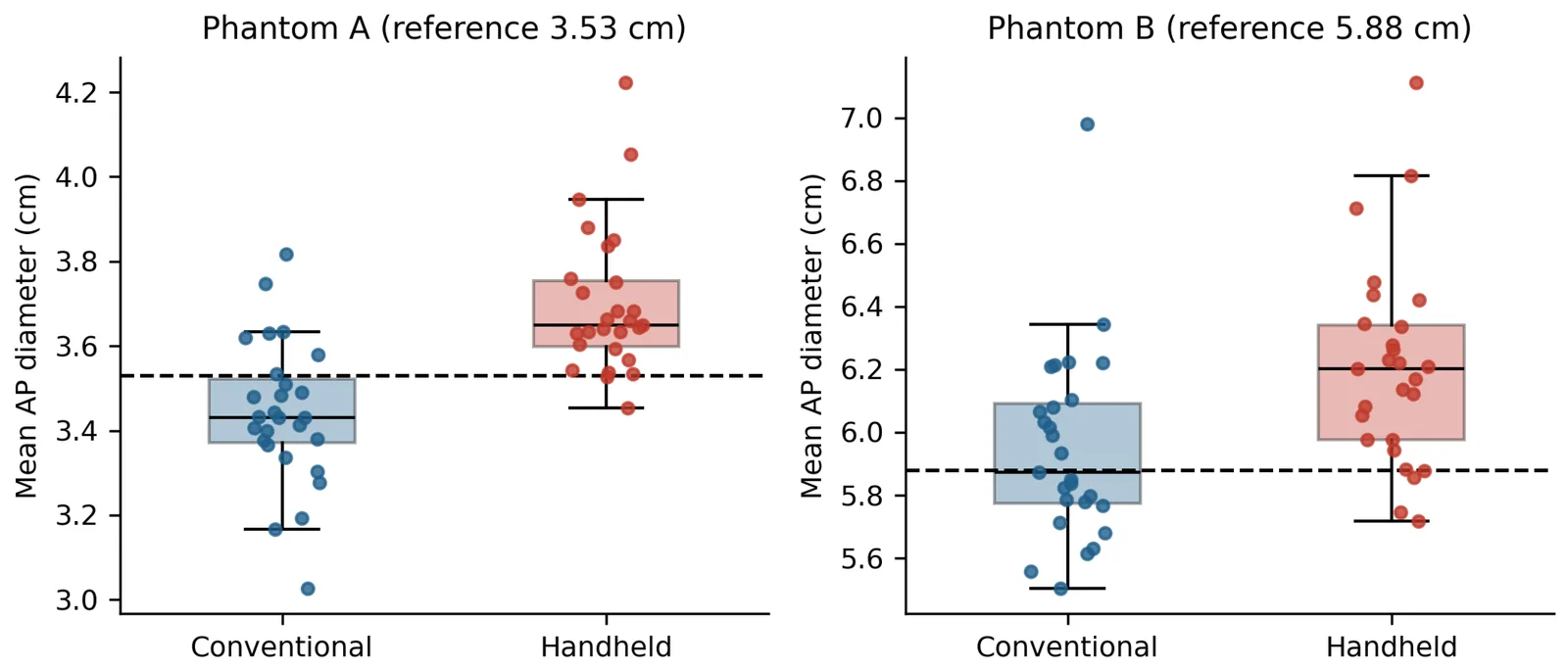
Distribution of per-participant mean anteroposterior diameters for Phantom A (**left**) and Phantom B (**right**) measured with the conventional and handheld systems. Boxes show the median and interquartile range, whiskers the 1.5 × IQR, points the individual participants, and the dashed line the pre-embedding calculated reference diameter.

Table 1 summarises participant means for each condition, and Figure 2 shows their distribution relative to the calculated reference values. On both phantoms, handheld measurements were higher than conventional measurements, and the spread of participant means (SD ≈ 0.17 cm on Phantom A and ≈0.31–0.32 cm on Phantom B; between-participant CV ≈ 5% in all conditions) was similar for the two systems.

### 3.3. Agreement with the Pre-Embedding Calculated Reference Diameter

**Figure 3:**
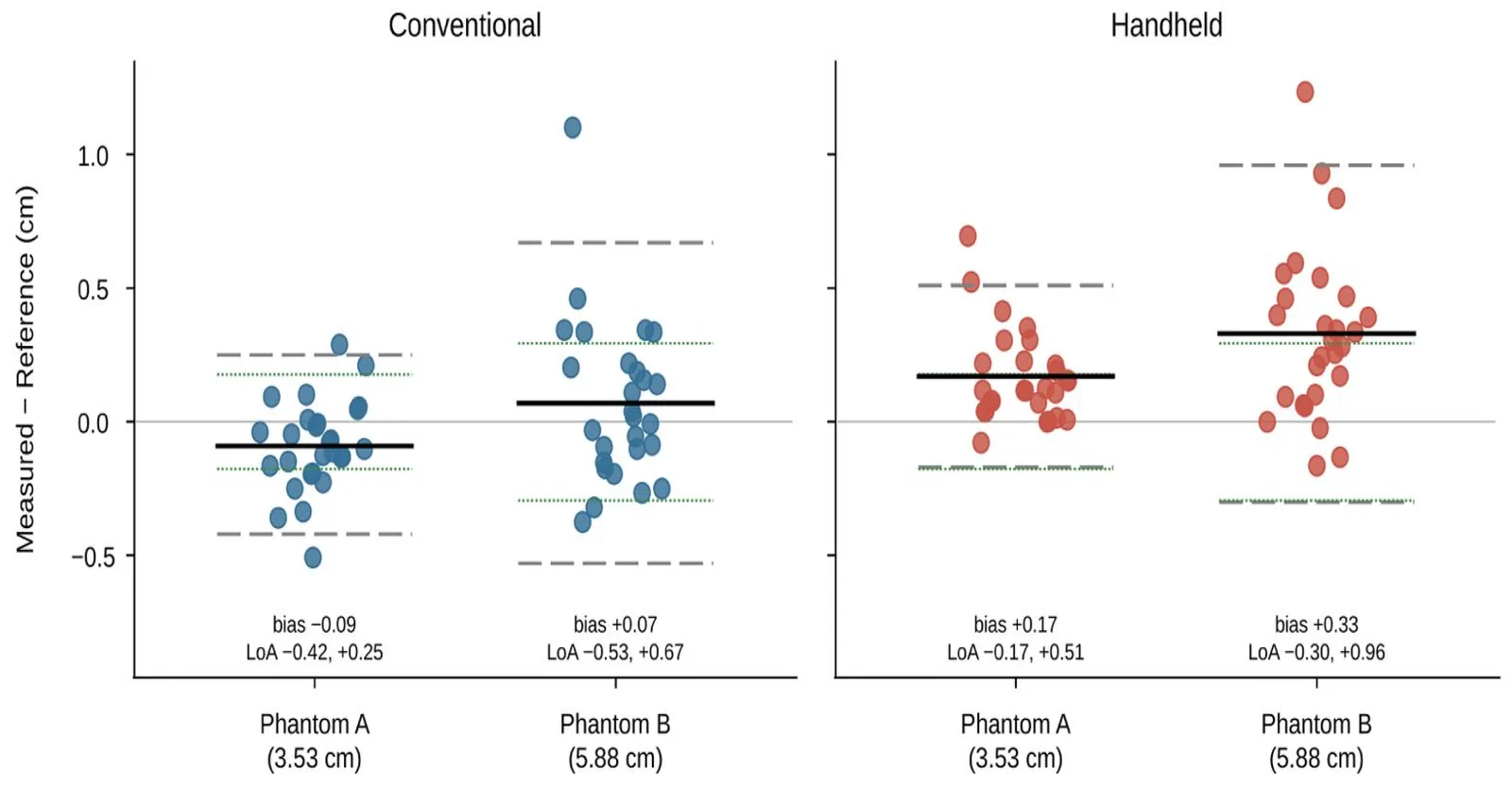
Difference plots of per-participant mean measurements against the pre-embedding calculated reference diameter for the conventional (**left**) and handheld (**right**) systems. Differences are plotted at the reference value of each phantom (with horizontal jitter for visibility), as recommended when the comparator is a fixed reference [33]. Solid line, mean bias; dashed lines, 95% limits of agreement; dotted green lines, the ±5% tolerance.

Table 2 and Figure 3 present bias, equivalence and agreement for each condition. All bias estimates are relative to the pre-embedding calculated reference (Section 2.2). With the conventional system, participants underestimated Phantom A by a mean of 0.09 cm (95% CI −0.16 to −0.02; −2.5%; *p* = 0.012) and overestimated Phantom B by a mean of 0.07 cm (95% CI −0.05 to +0.19; +1.2%; *p* = 0.25). The non-significant result for Phantom B cannot in itself be interpreted as evidence of no bias, given its wide 95% CI; however, the equivalence test showed that the conventional-system bias was statistically within the ±5% margin for both phantoms (TOST *p* = 0.007 and *p* < 0.001, respectively). The 90% CIs of the conventional-system bias were −4.1% to −0.9% (Phantom A) and −0.5% to +2.9% (Phantom B), so equivalence would also hold for any margin of ±4.1% or wider. With the handheld device, participants overestimated both phantoms by a mean of 0.17 cm (95% CI 0.10–0.24; +4.8%; *p* < 0.001) and 0.33 cm (95% CI 0.20–0.45; +5.6%; *p* < 0.001), effects of large, standardised size (dz = 0.99 and 1.02), and equivalence within ±5% could not be demonstrated for either phantom (TOST *p* = 0.42 and *p* = 0.70; 90% CIs +3.2% to +6.4% and +3.8% to +7.4%).

The 95% limits of agreement were of similar width for the two systems on each phantom (≈ 0.67 cm on Phantom A and ≈ 1.20–1.26 cm on Phantom B, i.e., about 19–21% of the reference diameter in every condition); the systems differed in the location of the interval rather than in its width (Figure 3). Mean absolute percentage error was 4.3% and 3.8% for the conventional system and 5.0% and 6.0% for the handheld device on Phantoms A and B, respectively. Expressed in absolute terms, MAD and RMSE were larger on Phantom B than on Phantom A for both systems, but this reflects the larger diameter: no clear difference in relative error was found between the two phantom sizes (Section 3.5).

### 3.4. Comparison Between Ultrasound Systems

**Figure 4:**
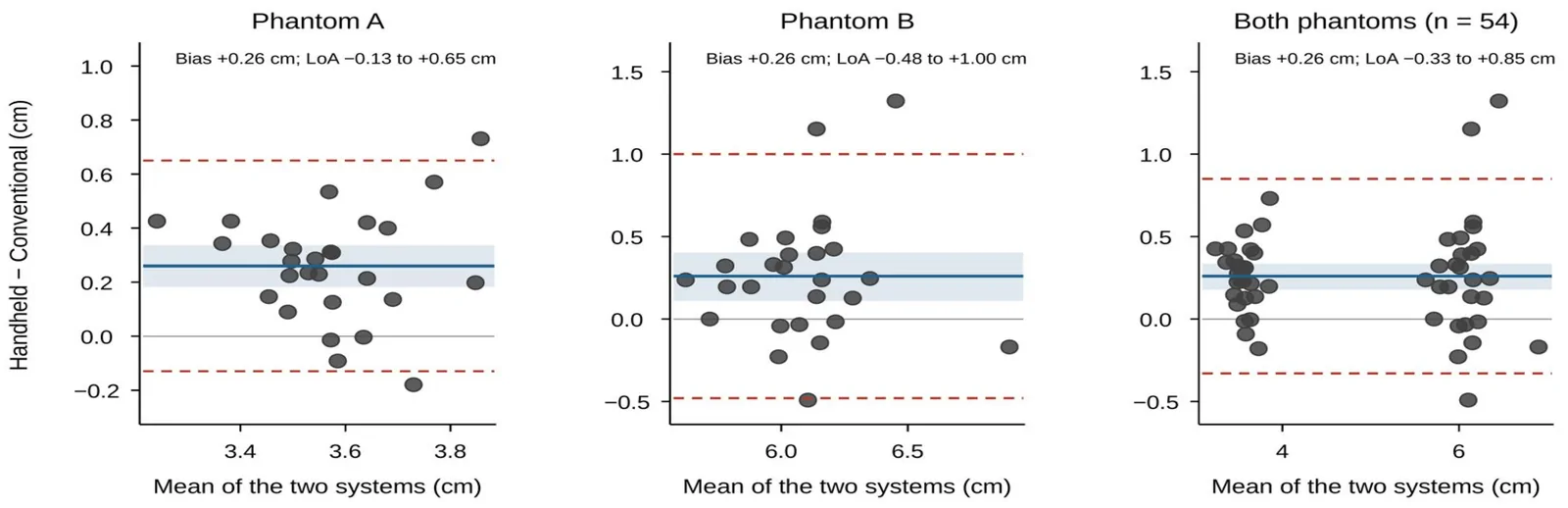
Bland–Altman plots of the difference between handheld and conventional per-participant means against the mean of the two systems for Phantom A (**left**), Phantom B (**centre**) and both phantoms pooled (**right**). Blue line and band: mean difference with its 95% CI; red dashed lines: 95% limits of agreement; grey line: zero difference.

Measurements obtained with the handheld device were systematically higher than those obtained with the conventional system by almost the same amount on both phantoms: +0.26 cm (95% CI 0.18–0.34; *p* < 0.001; dz = 1.29) on Phantom A and +0.26 cm (95% CI 0.11–0.41; *p* = 0.001; dz = 0.68) on Phantom B (Table 3). The Bland–Altman plots (Figure 4) show no proportional bias (regression of difference on mean: slope 0.015 cm/cm, *p* = 0.96 for Phantom A; 0.080 cm/cm, *p* = 0.79 for Phantom B; 0.001 cm/cm, *p* = 0.97 pooled), so, across the two phantom sizes studied, the between-system difference behaved as a constant offset rather than a percentage. The limits of agreement were wider for Phantom B (−0.48 to +1.00 cm) than for Phantom A (−0.13 to +0.65 cm), and 47 of the 54 paired differences (87%) were positive. The repeated-measures ANOVA on signed percentage error confirmed a large main effect of system (F(1,26) = 36.2, *p* < 0.001, ηG2 = 0.26), a smaller effect of phantom (F(1,26) = 14.9, *p* < 0.001, ηG2 = 0.047) that reflects the conventional system’s change from slight under- to slight overestimation between phantoms, and a small system × phantom interaction (F(1,26) = 5.2, *p* = 0.031, ηG2 = 0.021). Because the handheld device was always used second and was unfamiliar to the participants, these between-system differences reflect the combined effect of device, operator familiarity and measurement order.

### 3.5. Percentage-Error Classification and Effect of Aneurysm Size

**Figure 5:**
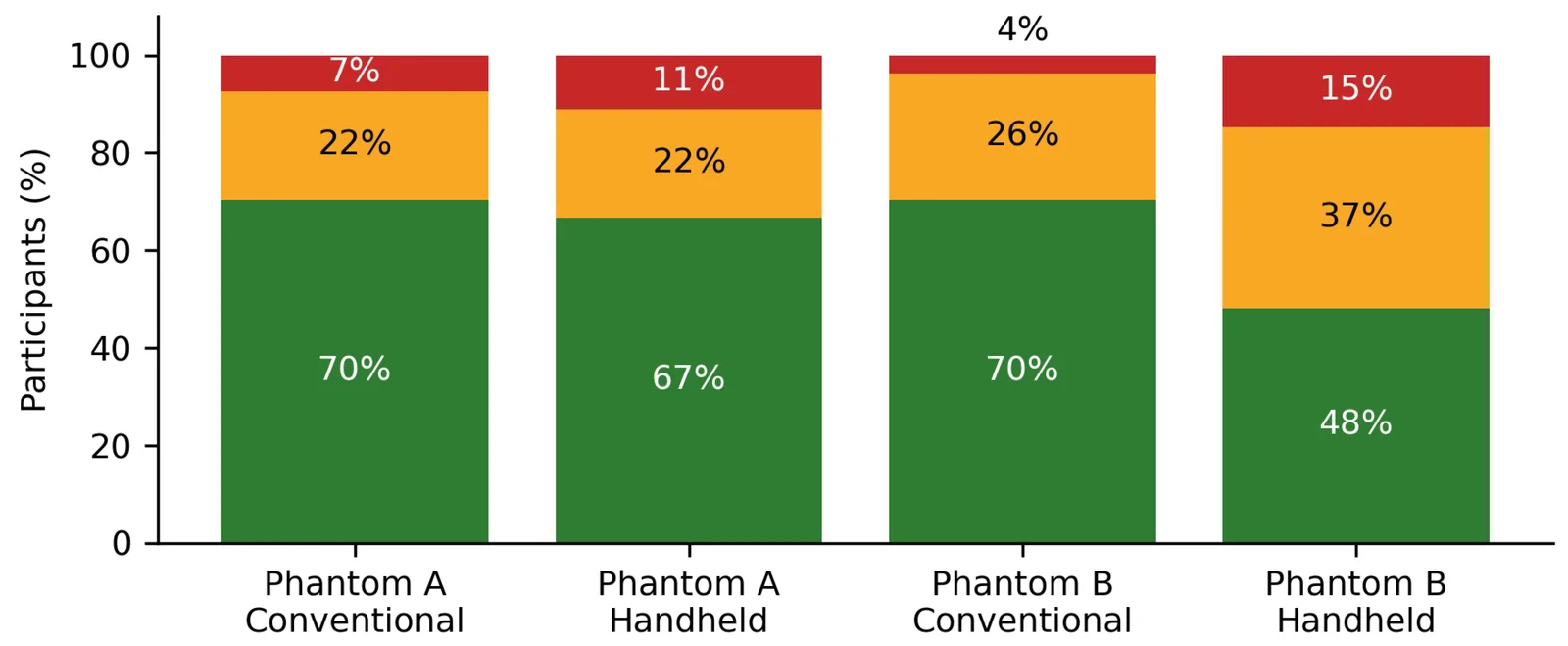
Percentage-error classification of per-participant mean measurements, relative to the pre-embedding calculated reference, for each phantom and ultrasound system.

Table 4 and Figure 5 show the classification of participant means by percentage difference from the calculated reference. With the conventional system, 70.4% of participant means were within 5% of the calculated reference on both phantoms, and 92.6% (Phantom A) and 96.3% (Phantom B) were within the 10% tolerance; pooled across phantoms, 51 of 54 conventional means (94.4%, 95% CI 85–98%) were within 10%. With the handheld device, 66.7% (Phantom A) and 48.1% (Phantom B) were within 5% and 88.9% and 85.2% were within 10% (pooled 47/54, 87.0%, 95% CI 76–94%). The between-system difference in the proportion of participant means within 5% was not statistically significant on either phantom (exact McNemar *p* = 1.00 for Phantom A, seven participants within 5% only with the conventional system versus six only with the handheld device; *p* = 0.15 for Phantom B, nine versus three). Seven of the 27 participants (26%) produced at least one mean with >10% error, and two participants did so in more than one condition.

None of the conventional-system means crossed a guideline surveillance threshold: all Phantom A means remained in the 3.0–3.9 cm band and all Phantom B means remained above 5.5 cm. With the handheld device, two participants (7.4%) recorded a Phantom A mean of ≥4.0 cm (4.05 and 4.22 cm), which, if observed in a patient, would correspond to the boundary between the three-year and one-year surveillance categories [5]; no handheld Phantom B mean fell below 5.5 cm. These threshold crossings occurred under idealised phantom conditions and are reported only as an illustration of the potential consequence of a systematic between-system difference, not as an estimate of clinical misclassification.

No clear difference in relative error was demonstrated between the two phantom sizes evaluated. In the repeated-measures ANOVA on absolute percentage error, neither the phantom effect (F(1,26) = 0.20, *p* = 0.66, ηG2 = 0.001) nor the system effect (F(1,26) = 1.92, *p* = 0.18) nor their interaction (F(1,26) = 1.72, *p* = 0.20) was significant, and a Friedman test across the four conditions was likewise non-significant (χ2(3) = 2.15, *p* = 0.54). Within each system, MAPE on Phantom B versus Phantom A was 3.8% versus 4.3% (conventional; Wilcoxon *p* = 0.17) and 6.0% versus 5.0% (handheld; *p* = 0.21).

### 3.6. Repeatability of Repeated Readings

Table 5 summarises the intra-observer repeatability of the three readings. Single-reading ICC(2,1) was 0.44 (95% CI 0.21–0.66) for Phantom A on the conventional system, 0.68 (0.48–0.82) for Phantom A on the handheld device, 0.79 (0.65–0.89) for Phantom B on the conventional system and 0.59 (0.37–0.76) for Phantom B on the handheld device; for the reasons given in Section 2.5, these values are not assigned to reliability categories and are secondary to the absolute indices below. Averaging three readings raised the ICC to 0.70–0.92, but the CIs of adjacent conditions overlapped substantially and the ranking of conditions was not the same as that of the absolute indices.

In absolute terms, the within-subject SD of a single reading ranged from 0.11 cm to 0.24 cm (within-subject CV 2.5–4.7%), and the repeatability coefficient, the maximum difference expected between two readings by the same participant on 95% of occasions, ranged from 0.31 cm (8.7% of the reference) for Phantom A on the handheld device to 0.67 cm (11.5%) for Phantom B on the handheld device. Thus the handheld device was more repeatable than the conventional system on the smaller phantom (0.31 vs. 0.45 cm) but less repeatable on the larger phantom (0.67 vs. 0.41 cm); neither system was uniformly more consistent. The largest range among an individual participant’s three readings was 0.98 cm (Phantom A, conventional) and 1.44 cm (Phantom B, handheld). Between-participant variability was of similar magnitude to within-participant variability on Phantom A (reproducibility coefficient ≈ 0.47–0.48 cm) and exceeded it on Phantom B (≈0.85–0.89 cm).

### 3.7. Temporal Stability of the Phantoms

**Figure 6:**
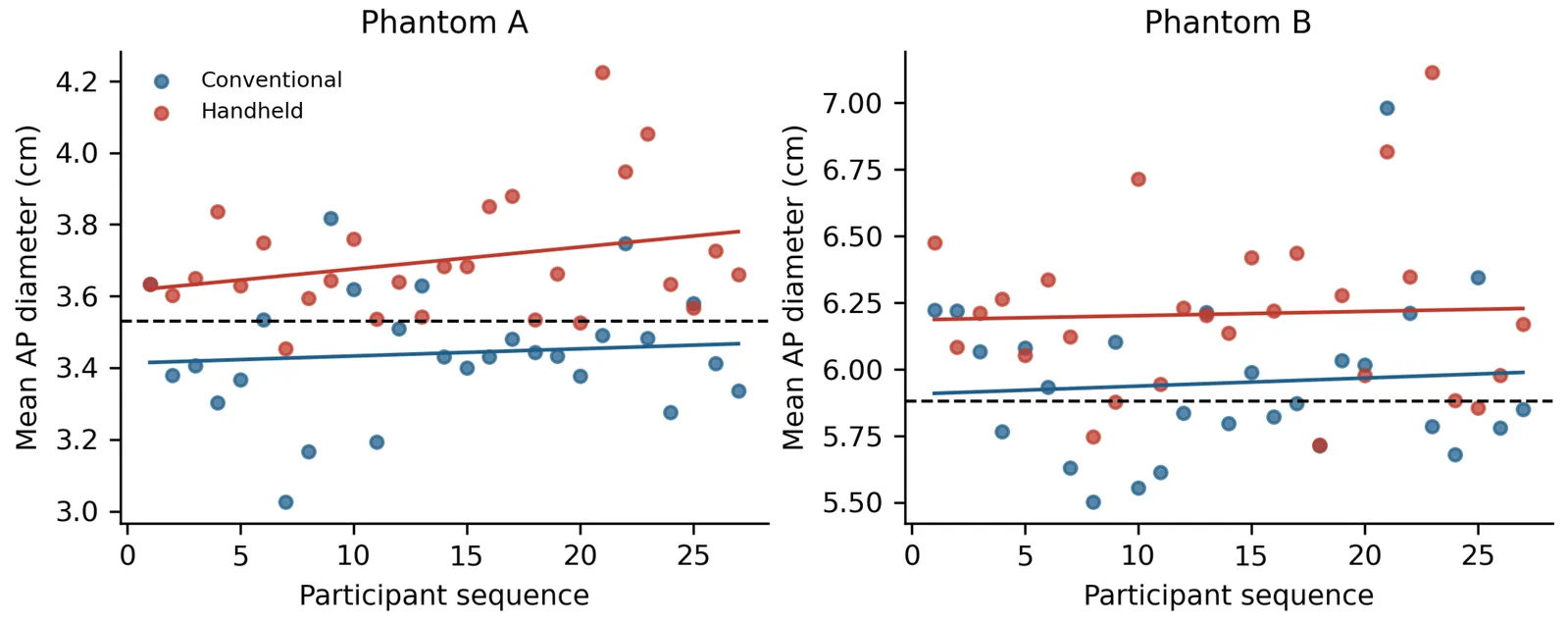
Per-participant mean anteroposterior diameter plotted against the chronological sequence of participant sessions for Phantom A (**left**) and Phantom B (**right**), with linear regression lines for the conventional and handheld systems. The dashed line is the pre-embedding calculated reference diameter.

No trend in measured diameter across the sequence of participant sessions was detected in any condition (Table 6, Figure 6). Regression slopes ranged from +0.002 to +0.006 cm per participant, all with 95% CIs including zero (*p* = 0.15–0.85); Spearman correlations were likewise non-significant (*p* = 0.26–0.88; not tabulated), and the first nine and last nine participants did not differ (*p* = 0.14–0.55). Any drift in the phantoms over the data-collection period was therefore smaller than the study could detect and, if present, was in the direction of a slight increase rather than the decrease expected from gelatin dehydration or balloon leakage.

## 4. Discussion

This phantom study quantified, against a pre-embedding calculated reference diameter, the agreement and repeatability with which senior sonography students measure an abdominal aortic aneurysm on a familiar conventional cart-based system and on an unfamiliar handheld device. Three findings stand out. First, on the conventional system, the students’ mean measurements were statistically equivalent to the calculated reference within a ±5% margin on both phantoms; 94% of participant means were within the 10% tolerance and none crossed a guideline surveillance threshold. Second, measurements on the unfamiliar handheld device, which was always used second, were consistently about 0.26 cm higher than those on the conventional system and 0.17–0.33 cm higher than the calculated reference; this difference was similar on the two phantom sizes studied and, in two participants, reached the 4.0 cm surveillance boundary on the smaller phantom. Because device type was confounded with operator familiarity and testing order, this difference cannot be attributed to the handheld device itself. Third, repeatability was condition-dependent rather than device-dependent: the handheld device was the more repeatable of the two on the smaller phantom and the less repeatable on the larger one, and single-reading repeatability coefficients of 0.31–0.67 cm indicate limited precision of individual readings.

### 4.1. Agreement with the Calculated Reference on the Conventional System

The conventional-system bias of −0.09 cm on the 3.53 cm phantom and +0.07 cm on the 5.88 cm phantom is small relative to the 0.3–0.5 cm inter-observer variability reported for aortic diameter in clinical settings [14,15,19,37] and well within the ±5% equivalence margin (Section 2.5) [30]. That the students underestimated the small phantom and slightly overestimated the large one suggests that the two errors have different sources. Underestimation of the 3.53 cm balloon is consistent with a small amount of flattening of the balloon’s anteroposterior dimension under the weight of the overlying gelatin layer: the reference diameter was derived from the circumference, which averages the anteroposterior and transverse dimensions, whereas the students measured only the anteroposterior dimension. Slight overestimation of the larger balloon is consistent with the well-described tendency of the OTO convention to include part of the wall echo, an effect that is proportionally larger when the wall is imaged at a shallower angle over a wider structure [16,17]. Neither effect exceeded 2.5% on average. Both explanations are hypothetical and, because the reference was calculated before embedding (Section 4.5), part of the apparent bias may reflect the reference itself rather than the operators. The comparison with clinical variability in the first sentence should also be interpreted cautiously because the phantoms presented an idealised target without the acoustic challenges of clinical scanning.

The proportion of students whose mean was within 5% of the calculated reference (70%) is comparable to that reported for medical students measuring aortic dimensions after focused training [12,13], and the 95% limits of agreement of about ±0.35 cm (Phantom A) and ±0.60 cm (Phantom B) are of the same order as the inter-observer limits reported by trained screening technicians in the UK programme [19] and by observers in the reproducibility study of Matthews et al., who found inter-observer reproducibility coefficients of 4–5 mm for the AP diameter [9]. The students’ between-participant reproducibility coefficients (0.47 cm on Phantom A and 0.85 cm on Phantom B) were somewhat wider than these clinical values, which is to be expected of operators at the end of undergraduate training measuring an unfamiliar object. The results therefore support a cautiously positive statement about the students’ measurement performance on the conventional system, but that statement must be qualified: one in four students produced at least one mean that exceeded the 10% tolerance, and the repeatability coefficient of a single reading on Phantom A (0.45 cm) is larger than the 0.4–0.5 cm difference that separates adjacent surveillance categories [5,6].

### 4.2. Between-System Difference: Device Characteristics, Operator Familiarity or Testing Order?

The most notable finding is the constant positive difference between the handheld and the conventional measurements under the conditions of this study. Its magnitude (0.26 cm) was the same on both phantoms, its 95% CI excluded zero by a wide margin, no proportional bias was detected, and 87% of participants showed a positive difference. We hypothesise that a constant difference of this kind is more likely to arise from a systematic difference in how the balloon wall was rendered and how the students placed the callipers on the handheld display than from random operator error, which would have widened the limits of agreement without shifting their centre. Three explanations are plausible; all are hypotheses that the present design could not test or distinguish. First, the offset may reflect a device-level characteristic: the lower spatial resolution and signal-to-noise ratio of handheld probes, which have fewer processing channels than cart-based consoles with dedicated hardware beamforming [21,22], together with the smaller display, may blur the outer boundary of the balloon wall, and under the OTO convention an operator who cannot resolve the wall edge tends to place the callipers beyond it. Second, the offset may reflect an operator-level effect of unfamiliarity: the students had no prior hands-on experience with the handheld device, whereas they had used the conventional system throughout their clinical placements; unfamiliarity with the calliper interface and gain settings of a new device can degrade image optimisation and measurement performance in novices [21,23]. Third, because the handheld device was always used second, a learning effect (for example, increasing familiarity with the phantoms) or a fatigue effect towards the end of a 12-measurement session may have contributed; the direction and size of such an order effect cannot be quantified in this design, and the similarity of the offset on both phantoms does not exclude it, because the order was the same for both phantoms. The data cannot differentiate between these explanations. It is equally possible that equivalent training on both systems would substantially narrow or eliminate the offset, in which case the 0.26 cm figure would reflect a training gap rather than a device characteristic. The offset should therefore not be interpreted as evidence that the handheld device is inherently inaccurate; what the data do show is that measurement competence acquired on a familiar system did not transfer automatically to an unfamiliar device after a brief orientation. Accordingly, the 0.26 cm figure is best described as an observed between-system difference under the conditions of this study, not as an intrinsic calibration bias of the handheld device.

Whether a difference of 0.26 cm matters depends on the clinical question. It is below the 0.4–0.5 cm inter-observer variability generally accepted for aortic ultrasound [14,15,37], it did not change the classification of the 5.88 cm phantom in any participant, and the systematic review by Rykkje et al. concluded that handheld devices detect AAA reliably [21]. For detection, therefore, the present findings are reassuring, although they were obtained on phantoms. For surveillance, however, an offset that is systematic rather than random is not diluted by averaging and would be carried into every serial measurement made on the device: two of 27 handheld means for the 3.53 cm phantom reached 4.0 cm, the boundary between three-yearly and yearly surveillance in current guidance [5]. In clinical practice, the consequence of such an error would not be a direct change in the operative decision, which rests on CTA, but an inappropriately shortened surveillance interval or a premature referral for CTA [5,6,8]. Moreover, this observation was made on a gelatin phantom without bowel gas, body habitus, mural thrombus or calcification and should not be extrapolated to a clinical misclassification rate. Bonnafy et al. reported that a pocket-sized device could be an appealing alternative for aortic diameter measurement by novices [11]; our data suggest that this presupposes adequate device-specific training and that any residual operator–device offset should be recognised, for example, by referencing follow-up measurements to the same device. This is consistent with the recommendation that serial aortic measurements should be made with a consistent method and, where possible, on the same equipment [5,14]. Accordingly, until the accuracy of trained operators on handheld devices has been established in patients, handheld systems are best used for rapid bedside detection and screening, whereas definitive diameter measurement for surveillance decisions should be performed on a cart-based system, and pre-operative planning and decisions on intervention should rely on CTA [5,6,8].

### 4.3. Repeatability

Cronbach’s α is sometimes used to summarise the consistency of repeated measurements, and on these data it would take values of 0.71–0.92 that would conventionally be labelled acceptable to excellent. Cronbach’s α is, however, a measure of the internal consistency of a multi-item psychometric scale, and although it is algebraically equal to the consistency-type average-measure ICC, it is insensitive to systematic differences between readings and its conventional thresholds were not developed for physical measurement [35]. We therefore report the absolute-agreement ICC with confidence intervals and, more importantly, the absolute indices that clinicians can relate to the measurement scale [31,32,34]. On these indices, single-reading ICCs (0.44–0.79) had wide and overlapping CIs, and the repeatability coefficients of 0.31–0.67 cm mean that two readings of the same phantom by the same student could differ by up to 3–7 mm before the difference would be considered unusual. These values are larger than the intra-observer repeatability coefficients of 2–3 mm reported for trained observers [9] and of the same order as inter-observer coefficients in screening programmes [19], and they indicate that a single reading by a student at this stage of training has limited precision. This limitation is not specific to students: a single reading by any single operator, trained or not, is generally considered insufficient for surveillance, and clinical protocols typically rely on standardised acquisition with repeated or averaged measurements or a second reader [9,19,38].

It is also important to interpret the ICC correctly in this design. Because every participant measured the same object, the between-participant variance that forms the numerator of the ICC consists of participant-specific offsets rather than genuine differences in aortic size. A high ICC here means that each participant reproduced his or her own reading closely relative to how much participants differed from one another; it does not mean that readings were close to the reference. The handheld device on Phantom A illustrates this: its ICC(2,1) of 0.68 and repeatability coefficient of 0.31 cm were the best of the four conditions, yet its bias of +0.17 cm was significant and it failed the equivalence test. Repeatability and agreement with the reference are separate properties, and a general claim that conventional ultrasound is “more consistent” is not supported by the absolute indices: the conventional system was more repeatable on the larger phantom and less repeatable on the smaller one.

### 4.4. Aneurysm Size

The larger MAD in centimetres on the 5.88 cm phantom might suggest that measurement error increases with aneurysm size. When error is expressed relative to the diameter, as it must be for a classification scheme defined in percentage terms, this interpretation is not supported for the two sizes studied: absolute percentage error did not differ between the phantoms in the repeated-measures ANOVA or in the Friedman test, MAPE on the conventional system was in fact slightly lower on the larger phantom, and the limits of agreement were about 19–21% of the reference diameter in every condition. The larger centimetre error on Phantom B is therefore consistent with a scaling effect. Because only two phantom sizes were studied, however, the absence of a detectable difference does not establish that relative error is independent of size across the clinically relevant AAA diameter range. What did change with size was the between-system agreement: the limits of agreement between the handheld and conventional systems were wider for Phantom B, and the handheld device’s repeatability coefficient doubled between the phantoms, which may suggest, as a hypothesis, that resolving the wall of a large, deep structure on the handheld display was more difficult than resolving that of a small one. This effect is specific to the handheld condition and thus to the combination of device, operator familiarity and measurement order, rather than a general effect of size.

### 4.5. Validity of the Reference and of the Phantoms

The reference diameter was derived from the balloon circumference before embedding, and its uncertainty (≈ 0.03 cm for a 0.1 cm tape-reading error) is an order of magnitude smaller than the biases and limits of agreement reported here. This figure reflects only the tape-reading error, not the geometric assumptions discussed below. The reference was not, however, re-verified after embedding or at the end of the data-collection period, and homemade gelatin phantoms are known to dehydrate and degrade over days to weeks at room temperature unless preserved [26]. The temporal analysis argues against a material change in the phantoms during the study, although it cannot validate the absolute reference. There was no trend in measured diameter across the chronological sequence of 27 participant sessions in any condition; the largest implied change over the whole period was +0.16 cm and was not significant, and any drift that did occur was upward, not in the direction expected from dehydration or water loss. Agreement between the conventional-system means and the reference cannot be used as independent validation, because those measurements are themselves one of the methods under evaluation. The sequence analysis has limited power (it could detect a linear trend of roughly 0.2 cm over the study with 80% power) and cannot exclude a step change, so the absence of drift is only partial reassurance. More fundamentally, the calculated reference assumes a circular balloon whose geometry did not change on embedding, whereas the participants measured the anteroposterior diameter of the embedded balloon; any flattening or deformation would make the two quantities differ. The reference should therefore be regarded as a pre-embedding calculated reference rather than a verified true diameter, and the absolute bias and equivalence estimates, including the finding of equivalence on the conventional system, carry this additional uncertainty. By contrast, the between-system difference is a paired comparison of two measurements of the same embedded balloon and does not depend on the reference value. This is identified as a limitation, and direct verification of the embedded anteroposterior diameter with an independent method before and after data collection is recommended for future work (Section 4.7).

### 4.6. Implications for Training

The findings have three practical implications for sonography education. First, the students’ performance on the system they had used throughout their training was close to the calculated reference; because there was no baseline measurement or comparison cohort, this cannot be attributed to the curriculum itself, but the one-in-four rate of >10% errors and the repeatability coefficients of up to 0.45 cm indicate that calliper-placement skills should continue to be reinforced, for example, through repeated measurement of phantoms of independently verified diameter with immediate feedback, an approach that has been shown to be dose-dependent in simulation-based ultrasound training [39]. Second, competence on a conventional system did not transfer automatically to a handheld device with which the students had no experience. Structured, device-specific training, including explicit instruction on identifying the outer wall on a low-resolution display, should precede any use of handheld devices for vascular measurement, consistent with recommendations for structured handheld-ultrasound curricula [21,40]. Although the present design cannot show that such training would remove the difference, the absence of automatic transfer is itself a strong argument for it. Third, because the between-system difference was systematic, programmes that introduce handheld devices should consider a simple calibration exercise on a phantom of independently verified diameter so that the bias of each operator–device combination is known to its users, in the same way that operator experience has been shown to affect measurement accuracy in other organs [41].

### 4.7. Limitations

Several limitations should be acknowledged. The most important is that device type was confounded with operator experience: participants had used the conventional system throughout their clinical placements but had received only a brief orientation to the handheld device. The design therefore cannot separate a genuine device-level characteristic (spatial resolution, display size, calliper interface) from an operator-level effect of unfamiliarity (for example, suboptimal gain settings or difficulty identifying the wall boundary on a smaller screen), and equivalent training on both systems might substantially narrow or eliminate the observed difference. Accordingly, the between-system difference should not be interpreted as a measure of the intrinsic accuracy of the handheld device. In addition, the phantoms, although tissue-mimicking, had an approximately uniform speed of sound and did not reproduce the depth, subcutaneous fat, phase aberration, acoustic clutter, pulsatility, wall calcification, mural thrombus, bowel gas or body habitus that complicate aortic measurement in patients, so absolute error in clinical practice is likely to be larger than reported here; the study describes operator and equipment performance under idealised conditions rather than clinical error. In patients, these factors blur the vessel margins and make it harder to distinguish the lumen, eccentric mural thrombus and the outer adventitial wall, which may affect a lower-resolution device disproportionately [21,22]; the threshold-crossing analysis in particular should be regarded as illustrative rather than as an estimate of clinical misclassification. Only two phantoms were used, so an inter-observer ICC could not be estimated, the effect of size rests on two levels, and the absence of a detectable size effect cannot be generalised across the clinically relevant AAA diameter range. Furthermore, the order of systems and phantoms was fixed for all participants (conventional first, handheld second; Phantom A before Phantom B), which introduces a learning or fatigue effect that cannot be quantified in this design; the similarity of the offset on both phantoms does not exclude such an effect, because the order was identical for both phantoms, and the offset therefore cannot be claimed to be purely device-related. The reference diameter was calculated from the balloon circumference before embedding, assuming a circular cross-section, and was not verified after embedding with an independent method; because the balloon may have become non-circular after embedding, the calculated reference and the embedded anteroposterior diameter may differ. The temporal-stability analysis shows only the absence of detectable change over time, and agreement of the conventional-system means cannot validate the reference. Absolute bias and equivalence estimates should therefore be interpreted as agreement with a calculated reference, whereas the between-system comparison is unaffected. The sample comprised the whole eligible cohort but was small (n = 27) and drawn from a single institution, and the 95% CIs for the conventional-system bias on Phantom B and for the size comparisons were correspondingly wide, so non-significant results in these comparisons should not be read as evidence of no effect. No expert sonographers were included as a comparison group, so the students’ performance is benchmarked against the reference and against published clinical variability rather than against local experts. Finally, only one handheld device and one conventional system were tested, and the between-system difference reported here should not be assumed to apply to other devices. Future studies should use a randomised or counterbalanced crossover design in which participants receive equivalent training on both systems, so that device-level and operator-level contributions can be separated.

## 5. Conclusions

On the conventional ultrasound system that they had used throughout their training, senior sonography students measured the anteroposterior diameter of two balloon-in-gelatin aneurysm phantoms with mean bias statistically equivalent to the pre-embedding calculated reference within a ±5% margin, with 94% of their means within 10% of the calculated reference and no measurement crossing a guideline surveillance threshold. On a handheld device with which they had no prior hands-on experience, and which was always used second, the same students’ measurements were systematically about 0.26 cm higher than on the conventional system and failed the equivalence test on both phantoms. Because device type was confounded with operator familiarity and with a fixed testing order, this is an observed between-system difference under the study conditions; it cannot be attributed to the handheld device itself and should not be regarded as an intrinsic calibration bias of the device. Single-reading repeatability coefficients were 0.31–0.67 cm and were not consistently better on either system, and no clear difference in relative error was demonstrated between the two phantom sizes evaluated.

These results indicate that, at the end of the programme’s ultrasound curriculum, students can measure the diameter of a simulated aneurysm in close agreement with a calculated reference on conventional equipment (although, without a baseline measurement or comparison cohort, this cannot be attributed to the curriculum itself), but that this competence cannot be assumed to transfer to an unfamiliar handheld device after only a brief orientation. The findings therefore underline the critical need for structured, device-specific training before handheld systems are used for aortic measurement, together with continued reinforcement of single-reading repeatability; whether such training narrows or eliminates the observed difference remains to be established. The implications of these findings are primarily educational and technical: in addition to such training, programmes that introduce a handheld device should check each trained user’s measurements on a phantom of independently verified dimensions. Clinical implications, such as the need to perform serial surveillance on a consistent system or the appropriate role of handheld devices in surveillance, cannot be established from a phantom study; pending evidence from patients, handheld devices are best regarded as tools for detection and screening, with definitive diameter measurement on a cart-based system and pre-operative decisions based on CTA. The findings are derived from a phantom model that does not reproduce clinical scanning conditions such as bowel gas and body habitus, one handheld device and a single-institution cohort, and they should be confirmed in patients, across several handheld platforms and with an expert comparison group before being generalised. Future studies should also verify the embedded anteroposterior diameter directly with an independent method before and after data collection, randomise or counterbalance the order of devices and phantoms, provide equivalent training on both systems, and include phantoms spanning the full surveillance range.

## Figures and Tables

**Table 1:** Descriptive statistics of the per-participant mean anteroposterior diameter (cm) for each phantom and ultrasound system (n = 27 participants per condition).

Phantom (Reference)	System	Mean ± SD	95% CI of Mean	Median [IQR]	Range	Shapiro–Wilk W (*p*)
A (3.53 cm)	Conventional	3.44 ± 0.17	3.37–3.51	3.43 [3.37–3.52]	3.03–3.82	0.979 (0.84)
A (3.53 cm)	Handheld	3.70 ± 0.17	3.63–3.77	3.65 [3.60–3.76]	3.45–4.22	0.884 (0.006)
B (5.88 cm)	Conventional	5.95 ± 0.31	5.83–6.07	5.87 [5.77–6.09]	5.50–6.98	0.901 (0.014)
B (5.88 cm)	Handheld	6.21 ± 0.32	6.08–6.33	6.20 [5.98–6.34]	5.72–7.11	0.942 (0.13)

SD, standard deviation; CI, confidence interval; IQR, interquartile range.

**Table 2:** Bias, equivalence and agreement of per-participant mean measurements relative to the pre-embedding calculated reference diameter.

Phantom/System	Bias, cm (95% CI)	Bias, %	One-Sample t (*p*) ^a^	d_z_ ^b^	TOST *p* (±5%) ^c^	95% LoA, cm	MAD ± SD, cm	MAPE, %	RMSE, cm
A/Conventional	−0.089 (−0.157 to −0.021)	−2.5	−2.71 (0.012)	−0.52	0.007	−0.42 to +0.25	0.152 ± 0.116	4.3	0.19
A/Handheld	+0.170 (+0.102 to +0.238)	+4.8	5.12 (<0.001)	0.99	0.42	−0.17 to +0.51	0.176 ± 0.166	5.0	0.24
B/Conventional	+0.069 (−0.052 to +0.190)	+1.2	1.17 (0.25)	0.23	<0.001	−0.53 to +0.67	0.225 ± 0.213	3.8	0.31
B/Handheld	+0.328 (+0.201 to +0.455)	+5.6	5.30 (<0.001)	1.02	0.70	−0.30 to +0.96	0.352 ± 0.294	6.0	0.45

^a^ df = 26. ^b^ Cohen’s d_z_. ^c^ Two one-sided tests of equivalence within ±5% of the calculated reference (±0.18 cm for Phantom A, ±0.29 cm for Phantom B); *p* < 0.05 indicates that the bias is statistically within the margin. LoA, limits of agreement; MAD, mean absolute deviation; MAPE, mean absolute percentage error; RMSE, root-mean-square error. Wilcoxon signed-rank *p*-values (not tabulated) were 0.012, <0.001, 0.39 and <0.001 for the four conditions, respectively, and led to the same conclusions as the one-sample *t*-tests.

**Table 3:** Paired comparison and Bland–Altman agreement between the handheld and conventional systems (difference = handheld − conventional).

Phantom	n	Mean Difference, cm (95% CI)	SD of Diff.	Paired t (*p*) ^a^	d_z_	Lower LoA (95% CI)	Upper LoA (95% CI)	Prop. Bias Slope (*p*)
A	27	+0.259 (0.180 to 0.339)	0.201	6.70 (<0.001)	1.29	−0.13 (−0.27 to 0.00)	+0.65 (0.52 to 0.79)	0.015 (0.96)
B	27	+0.259 (0.109 to 0.408)	0.378	3.55 (0.001)	0.68	−0.48 (−0.74 to −0.22)	+1.00 (0.74 to 1.26)	0.080 (0.79)
Pooled	54	+0.259 (0.177 to 0.341)	0.300	6.34 (<0.001)	0.86	−0.33 (−0.47 to −0.19)	+0.85 (0.71 to 0.99)	0.001 (0.97)

^a^ df = 26 (Phantom A, B) or 53 (pooled). LoA, 95% limits of agreement. Wilcoxon signed-rank *p*-values (not tabulated) were <0.001 (Phantom A), 0.001 (Phantom B) and <0.001 (pooled).

**Table 4:** Classification of per-participant mean measurements by absolute percentage difference from the pre-embedding calculated reference, n (%).

Category	Phantom A Conventional	Phantom A Handheld	Phantom B Conventional	Phantom B Handheld
≤5%	19 (70.4)	18 (66.7)	19 (70.4)	13 (48.1)
>5% to ≤10%	6 (22.2)	6 (22.2)	7 (25.9)	10 (37.0)
>10%	2 (7.4)	3 (11.1)	1 (3.7)	4 (14.8)
Within 10% tolerance	25 (92.6)	24 (88.9)	26 (96.3)	23 (85.2)
Crossing a surveillance threshold ^a^	0 (0.0)	2 (7.4)	0 (0.0)	0 (0.0)
McNemar *p*, ≤5% vs. >5% ^b^	1.00		0.15	

^a^ Mean ≥ 4.0 cm or < 3.0 cm for Phantom A; <5.5 cm for Phantom B. ^b^ Exact McNemar test comparing the conventional and handheld classification (≤5% vs. >5%) within the phantom.

**Table 5:** Intra-observer repeatability of the three repeated readings for each phantom and ultrasound system (n = 27 participants, 81 readings per condition).

Phantom/System	ICC(2,1) (95% CI)	ICC(2,3) (95% CI)	SD_w_, cm	CV_w_, %	Repeatability Coefficient, cm (% of Reference)	Max. Within-Participant Range, cm	SD_between_, cm	Reproducibility Coefficient, cm
A/Conventional	0.44 (0.21–0.66)	0.70 (0.45–0.85)	0.162	4.7	0.45 (12.7)	0.98	0.171	0.47
A/Handheld	0.68 (0.48–0.82)	0.86 (0.74–0.93)	0.111	3.0	0.31 (8.7)	0.64	0.173	0.48
B/Conventional	0.79 (0.65–0.89)	0.92 (0.85–0.96)	0.149	2.5	0.41 (7.0)	0.59	0.305	0.85
B/Handheld	0.59 (0.37–0.76)	0.81 (0.64–0.91)	0.243	3.9	0.67 (11.5)	1.44	0.321	0.89

ICC(2,1), two-way random-effects, absolute-agreement, single-reading intraclass correlation coefficient; ICC(2,3), the same model for the mean of three readings; SD_w_, within-subject SD; CV_w_, within-subject coefficient of variation; repeatability coefficient = 1.96 × √2 × SD_w_; SD_between_, SD of participant means; reproducibility coefficient = 1.96 × √2 × SD_between_.

**Table 6:** Linear trend in per-participant mean diameter across the chronological sequence of participant sessions, as a check on phantom stability.

Phantom/System	Slope, cm per Participant (95% CI)	Implied Change over 27 Sessions, cm	Pearson r (*p*)	Mean, First 9 vs. Last 9 Participants, cm (*p*)
A/Conventional	+0.0020 (−0.0068 to +0.0108)	+0.05	0.09 (0.64)	3.40 vs. 3.46 (0.55)
A/Handheld	+0.0061 (−0.0025 to +0.0147)	+0.16	0.28 (0.15)	3.64 vs. 3.78 (0.14)
B/Conventional	+0.0030 (−0.0128 to +0.0188)	+0.08	0.08 (0.70)	5.95 vs. 6.08 (0.43)
B/Handheld	+0.0016 (−0.0151 to +0.0182)	+0.04	0.04 (0.85)	6.13 vs. 6.27 (0.41)

## Data Availability

The data presented in this study are available on request from the corresponding author.

## Abbreviations

The following abbreviations are used in this manuscript:

AAA	abdominal aortic aneurysm
ANOVA	analysis of variance
AP	anteroposterior
CI	confidence interval
CTA	computed tomography angiography
CV	coefficient of variation
dz	Cohen’s d for paired differences
ICC	intraclass correlation coefficient
IQR	interquartile range
ITI	inner-to-inner
LELE	leading-edge-to-leading-edge
LoA	limits of agreement
MAD	mean absolute deviation
MAPE	mean absolute percentage error
OTO	outer-to-outer
RMSE	root-mean-square error
SD	standard deviation
TOST	two one-sided tests (equivalence testing)
